# Rapid Synthesis of Kaolinite Nanoscrolls through Microwave Processing

**DOI:** 10.3390/nano12183141

**Published:** 2022-09-10

**Authors:** Md Shahidul Islam Khan, John B. Wiley

**Affiliations:** Department of Chemistry and Advanced Materials Research Institute, University of New Orleans, New Orleans, LA 70148, USA

**Keywords:** kaolinite, nanoscrolls, microwave processing, rapid synthesis

## Abstract

Kaolinite nanoscrolls (NScs) are halloysite-like nanotubular structures of great interest due to their ability to superimpose halloysite’s properties and applicability. Especially attractive is the ability of these NScs to serve as reaction vessels for the uptake and conversion of different chemical species. The synthesis of kaolinite NScs, however, is demanding due to the various processing steps that lead to extended reaction times. Generally, three intercalation stages are involved in the synthesis, where the second step of methylation dominates others in terms of duration. The present research shows that introducing microwave processing throughout the various steps can simplify the procedure overall and reduce the synthesis period to less than a day (14 h). The kaolinite nanoscrolls were obtained using two final intercalating agents, aminopropyl trimethoxy silane (APTMS) and cetyltrimethylammonium chloride (CTAC). Both produce abundant NScs, as corroborated by microscopy measurements as well as the surface area of the final products; APTMS intercalated NScs were 63.34 m^2^/g, and CTAC intercalated NScs were 73.14 m^2^/g. The nanoscrolls averaged about 1 μm in length with outer diameters of APTMS and CTAC intercalated samples of 37.3 ± 8.8 nm and 24.9 ± 6.1 nm, respectively. The availability of methods for the rapid production of kaolinite nanoscrolls will lead to greater utility of these materials in technologically significant applications.

## 1. Introduction

Kaolinite nanoscrolls (NScs) are well known tubular structures that can be synthesized from bulk kaolinite. The kaolinite structure is layered and consists of two different sets of sheets, octahedral alumina, AlO_6_, and tetrahedral silica, SiO_4_, in 1:1 ratio [1,2,3,4]. Halloysite nanotubes, which are compositionally similar to kaolinite, have been investigated due to their potential applications in drug delivery [5], catalyst supports [6], waste water treatment [7], composite materials [8], and host structures for the preparation of nanocomposites [9,10,11]. Halloysites have limitations with respect to occurrence and purity, which creates a need for a divergent material, which can have properties such as these natural nanotubes [12,13]. Kaolinite NScs are good candidates for this replacement [14] and have the added benefit of structure tunability [15].

Singh and coworkers first reported methods for the synthesis of kaolinite NScs [16]. They showed that NScs could be synthesized by the repeated intercalation of kaolinite with potassium acetate followed by washing with excess water. Additionally, their determination that scrolls form along the b axis of the parent product is significant. More recently, Kuroda et al. reported a one-step route to produce kaolinite NScs, though this synthesis contains a long 7-day methylation step [17]. Later, Li et al. executed a different approach where they enhanced the intercalation of organics by an ultrasonication process [18]. Mako et al. reported a solvothermal process to obtain NScs from methylated kaolinite using cetyltrimethylammonium chloride (CTAC) as the final intercalating agent [19]. 

Despite their involved synthesis procedures, kaolinite NScs are still of interest with widespread applications. Nakagaki et al. synthesized an efficient and selective heterogeneous catalyst by immobilizing iron porphyrins within the kaolinite tubular structure [20]. Tang et al. synthesized a flame-retardant nanocomposite with polypropylene fused with kaolinite nanoscrolls [21]. Further, Abukhadra et al. successfully utilized kaolinite NScs to adsorb heavy metals from water [22]. 

A general procedure for synthesizing kaolinite NScs can be gleaned from the series of previously reported experiments [17,18,19,23]. Mainly, these NScs form in a three-step process, where the first step involves intercalated kaolinite products through polar molecule intercalation. The structure of kaolinite due to the presence of silica tetrahedral sheets (negatively charged) and alumina octahedral sheets (positively charged) possesses permanent dipolar characteristics, which favors the intercalation of small polar molecules such as dimethyl sulfoxide (DMSO) and N-methylforamide (NMF) [24,25]. This resulting intercalated structure allows for the polar methanol molecule to subsequently intercalate into the layers with displacement of the DMSO or NMF. Dettelier et al. pioneered methylation in the kaolinite structure [26]. This methylation is crucial in that without it, the formation of kaolinite NScs does not readily occur, and this step allows other organic molecules to intercalate into the kaolinite. In a third step, CTAC or amino propyl trimethoxy silane (APTMS) can then be used as scrolling agents leading to the targeted NScs [17,18,19]. The duration of the first and third steps are short compared to the methylation of intercalated kaolinites. Recently, Qu et al. were able to decrease the methylation processing time to 8 h through a Soxhlet separation method though the total required time to obtain the kaolinite nanoscrolls was 72 h [27].

The synthesis of kaolinite NScs has been done through various processes such as stirring [17], ultrasonication [18], solvothermal [19], etc. The microwave processing of the kaolinite material has been described by some researchers. Zhenbang et al. reported the exfoliation of kaolinite from urea-intercalated precursor with microwave heating [28], and Zhang et al. reported synthesis of kaolinite nanocomposites, where they treated kaolinite with polyethylene glycol followed by DMSO [29]. Mo et al. also employed a microwave for the modification of kaolinite to make a hydrophobic material with an organosilane, which later was utilized for oil recovery [30]. Zsirka et al. traced broken and cracked tubular structures where chip-like particles dominated during the synthesis of kaolinite nanocomposites [31]. Recently, Zhang et al. compared traditional heating with microwave heating during the study of the activation properties of zeolite precursors from kaolinite [32]. 

The importance of kaolinite nanoscrolls supports the need for faster and more streamlined processing of these materials. Microwave processing is known to shorten the reaction time compared to existing processes. In a recent article from Akbarian-Tefaghi et al., it was shown that microwave treatments could complete week-long reactions within hours [33]. Herein, we report on the synthesis of kaolinite NScs through multistep microwave processing. This approach not only shortens the reaction times for the production nanoscrolls from several days to half a day (14 h) but also simplifies the overall preparation. The products resulting from this method are of high quality and exhibit well defined tubular morphologies with an increased surface area.

## 2. Experimental

*Materials.* Powdered kaolinite (KGa-1b), originally sourced from Washington County, Georgia, USA, was purchased from the Clay Minerals Society. Before using the kaolinite, it was passed through a 200-mesh sieve. Dimethyl sulfoxide (DMSO, ACS reagent grade) was purchased from J. T. Baker (Phillipsburg, NJ, USA). N-methylformamide (NMF, 99%), methanol (99.8%, anhydrous), cetyltrimethylammonium chloride (CTAC, 98%), and (3-aminopropyl)trimethoxysilane (APTMS, 97%) were all obtained from Sigma Aldrich (St. Louis, MO, USA). Toluene (ACS grade) and ethanol (anhydrous, ACS grade) were acquired from Fisher Scientific (Fair Lawn, NJ, USA) and PHARMCO-AAPER (Brookfield, CT, USA) respectively.

*Microwave processing*. The different intercalation reactions utilized during the synthesis of kaolinite nanoscrolls generally take days. Microwave synthesis procedures can accelerate these reactions to obtain the desired products in a short amount of time. In the current procedure, the reactions were carried out in Milestone START microwave system (Milestone, Shelton, CT, USA). The system holds up to 32 individually capped reaction tubes (glass tubes can withstand pressures up to 15 bar). Each tube can hold 4–16 mL of solution and a magnetic stir bar coated with Teflon. If the reaction medium is nonpolar then a Weflon (graphite-doped Teflon) button is inserted inside the tube, which assists in the absorption of microwave radiation. ***Caution:*** Glass microwave reaction tubes need to be free from cracks and dirt; such imperfections can produce hot spots, possibly leading to explosions. The selection of reaction parameters should consider solvent boiling points and the pressure limitations of the reaction vessels. 

*Synthesis of intercalated kaolinite.* In total, 0.1 g kaolinite was placed in a microwave tube along with 1.0 mL deionized water and either 9.0 mL DMSO or 9.0 NMF. The volume of DMSO and NMF was selected by maintaining a ratio with water (9:1). The ratio was chosen by following Qu et al. and Olejnik et al.’s procedure during intercalation with DSMO and NMF, respectively [27,34]. The mixture was sonicated for 1 min and then heated in the microwave oven at 100 °C and 500 W for 5 h with magnetic stirring. After intercalation, the room temperature mixture was transferred to a 50 mL centrifuge tube and washed one time with 10.0 mL anhydrous methanol. The supernatant was discarded, and powder kept wet in methanol for the next step. The samples were labeled as KD for DMSO intercalated kaolinite and KN for NMF intercalated kaolinite.

*Methylation of intercalated kaolinite.* Wet KD or KN were dispersed in 10.0 mL anhydrous methanol and transferred from a centrifuge tube to a clean microwave tube. Then, the dispersed wet powder was sonicated for 10 min and combined with a magnetic stir bar. The sample was heated in the microwave for 1 h at 75 °C at 500 W with stirring. When the sample reached room temperature, the magnetic bar was removed. The process was repeated two more times with fresh anhydrous methanol. Finally, the sample was centrifuged in a 50 mL centrifuged tube. The two samples, KD and KN, were then called KM-1 or KM-2, respectively.

*Synthesis of kaolinite nanoscrolls (NScs).* Kaolinite NScs were obtained by two different routes. In one route, NScs were obtained through intercalation with APTMS and in another route, CTAC was intercalated.

*APTMS.* Wet KM-1 or KM-2 was placed in a microwave tube with one magnetic stirring bar, one Weflon button, and 10.0 mL APTMS. The intercalation was done at 90 °C at 500 W for 4 h. The sample was then washed 3 times with ethanol. The powder was collected and dispersed in 10 mL toluene and sonicated for 10 min. The sonication with fresh toluene was repeated three more times. Then the sonicated sample centrifuged, and the final sample stored in a glass vial.

*CTAC.* Wet KM-1 or KM-2 was placed in a microwave tube with 10 mL 1 M CTAC solution in methanol and then kept in a microwave with magnetic stirring for 4 h at 75 °C at 500 W. After the reaction, the sample was washed 3 times with ethanol. The powder was collected and dispersed in 10 mL toluene and sonicated for 10 min. The sonication with fresh toluene was repeated for three more times. Then, the sonicated sample was centrifuged and the final sample was stored in a glass vial.

*Characterization*. Samples were collected after each step during the synthesis of kaolinite NScs. Wet samples were dried in an oven at 70 °C for 24 h before characterization. The sample for X-ray powder diffraction (XRD) was mounted on a glass slide, and characterization was carried between 2.5 and 35.0° 2θ on a Rigaku Miniflex II Desktop X-ray diffractometer (Rigaku, Tokyo, Japan) with a scan speed of 0.75°/s; the instrument used CuKα radiation at 30 kV and 15 mA. A Hitachi 4800 High-resolution SEM (Hitachi, Tokyo, Japan) (Tulane University) was used to observe morphologies; the samples were carbon-coated before SEM imaging with a Cressington 208 HR sputter coater. For TEM imaging, dilute suspensions of powdered samples were prepared by dispersion in toluene via a 30 s sonication. Then, the suspension was drop cast on a Cu TEM grid (Ted Pella) and dried in an oven at 70 °C for 12 h. Transmission electron microscope (TEM) imaging was carried out on a JEOL 2010 TEM (JEOL, Peabody, MA, USA) at 200 kV and 110 mA. FTIR characterization utilized a Perkin Elmer System 2000 FTIR spectrometer (Perkin Elmer, Beaconsfield, England) where the sample was spread on a clean polished KBr salt plate (25 mm in diameter). For adsorption and desorption characterization, a Micromeritics ASAP 2020 (Micromeritics, Norcross, GA, USA) instrument was used with nitrogen gas. The sample was degassed at 300 °C for 30 min to remove moisture or other gases that previously adsorbed on the surface. The specific surface area of the sample was calculated from the BET theorem [35]. Additionally, the porosity of raw kaolinite and nanoscrolls was studied using the Barrett, Joyner, and Halenda (BJH) desorption dV/dD pore volume method [35]: dV represents distribution the curves of pore volume as a function of pore diameter and dD, the adsorption–desorption isotherm of nitrogen.

## 3. Results

A three-step procedure utilizing microwave methods has been developed that reduces processing times and streamlines the production of kaolinite NScs. Detailed crystallographic, vibrational, microscopic, and gas-adsorption studies on intermediates and products highlight the effectiveness of this approach. The rapidity of this method reduces processing times from 3 days to almost a ½ day.

**Figure 1:**
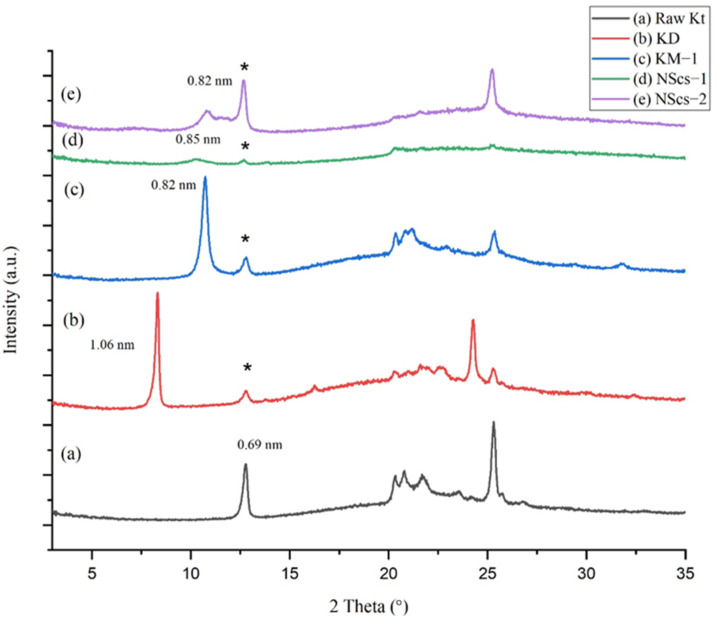
XRD patterns of (**a**) untreated kaolinite (Kt), (**b**) DMSO treated kaolinite (KD), (**c**) methanol treated kaolinite (KM-1), (**d**) APTMS intercalated nanoscrolls (NScs-1), and (**e**) CTAC intercalated nanoscrolls (NScs-2). (The asterisk (*) indicates unreacted kaolinite in the sample.).

The unreacted kaolinite parent (KGa-1b) shows the 001 plane corresponding to a d-spacing of 0.69 nm (Figure 1). Intercalation with DMSO, a step mandatory for further reaction in the structure, increases the spacing; DMSO intercalated kaolinite (KD) has a d-spacing of 1.06 nm. The increase in basal spacing of KD is 0.37 nm relative to kaolinite. Following intercalation with DMSO, methylation allows methanol to intercalate (graft) between the layers of kaolinite reducing the basal spacing to 0.82 nm due to the smaller size of methanol compared to that of the DMSO (Δ = 0.13 nm relative to parent). Then, the intercalation of APTMS in the methoxy-grafted kaolinite leads to scrolling; the nanoscroll sample, NScs-1, exhibits a broad and diminished peak corresponding to ~0.85 nm (Figure 1c). Nanoscrolls were also accessible by intercalation with CTAC (NScs-2) with d-spacing 0.82 nm. The reflection seen at 0.69 nm d-spacing is attributed to unreacted kaolinite. 

**Figure 2:**
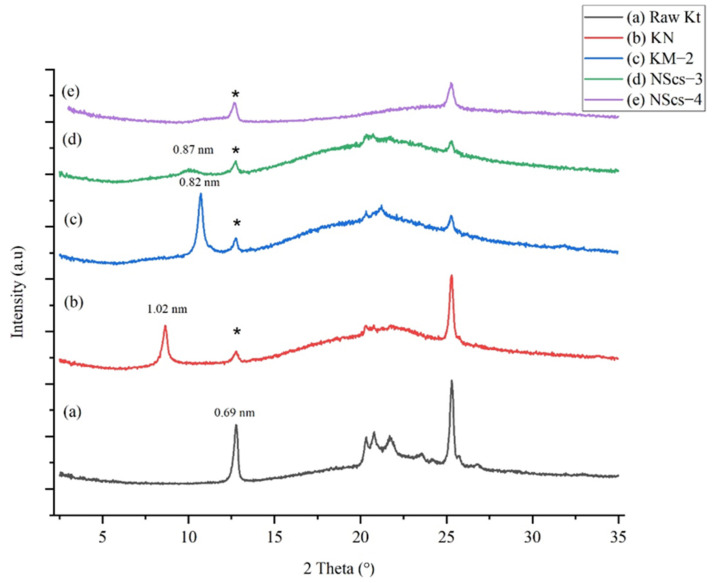
XRD pattern of (**a**) untreated kaolinite (Kt), (**b**) NMF treated kaolinite (KN), (**c**) methanol treated kaolinite (KM-2), (**d**) APTMS intercalated nanoscrolls (NScs-3), and (**e**) CTAC intercalated nanoscrolls (NScs-4). (The asterisk (*) indicates unreacted kaolinite in the sample.).

Another primary intercalating agent, NMF, was investigated. NMF also inserts into the layers of kaolinite and allows for subsequent methanol intercalation. XRD characterization (Figure 2) shows that the NMF intercalated kaolinite, KN, exhibits the 001-reflection corresponding to a d-spacing of 1.02 nm, which shows an increase in basal spacing of 0.33 nm. Following intercalation with methanol, the structure of the basal spacing is again reduced to 0.82 nm. After final reaction and scrolling from APTMS treatment (NScs-3), a broad peak is observed at 0.87 nm (Figure 2d). In the case of CTAC (NScs-4), however, no reflection is observed in that region (Figure 2e). Evidence for kaolinite is again observed at 0.69 nm.

Following the methods of Qu et al. [27], we can estimate the intercalation ratio in percent from XRD data for both the DMSO and NMF pathways to give an estimate of the percent unreacted kaolinite. This approach is based on fractional intensity ratios (intercalate/(intercalate + kaolinite)) of the 001 reflections for the intercalate products relative to kaolinite. In the case of DMSO intercalation, the ratio is found to be 83.0%, with the subsequent methanol intercalation at 78.7%. For NMF, the percent intercalation is not as extensive: 68.0% NMF intercalation and 68.7% subsequent methanol intercalation. Based on these calculations, we can roughly estimate that for DMSO, about 79% of the kaolinite participated in the reaction and for NMF only about 68%. 

**Figure 3:**
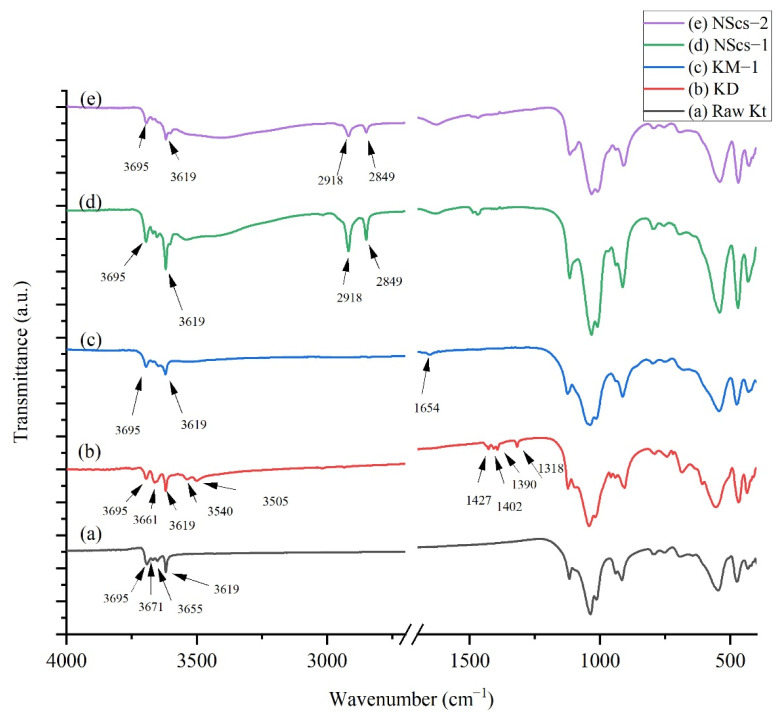
IR spectra of (**a**) untreated kaolinite (Kt), (**b**) DMSO treated kaolinite (KD), (**c**) methanol treated kaolinite (KM-1), (**d**) APTMS intercalated nanoscrolls (NScs-1), and (**e**) CTAC intercalated nanoscrolls (NScs-2).

**Figure 4:**
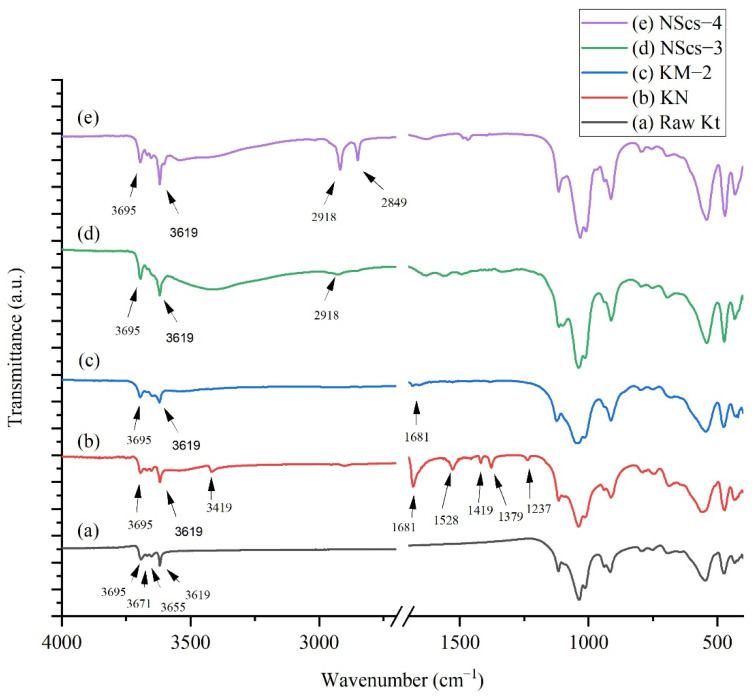
IR spectra of (**a**) untreated kaolinite (Kt), (**b**) NMF treated kaolinite (KN), (**c**) methanol treated kaolinite (KM-2), (**d**) APTMS intercalated nanoscrolls (NScs-3), and (**e**) CTAC intercalated nanoscrolls (NScs-4).

For IR analysis of kaolinites, different intermediate and nanoscrolls are shown in Figure 3 and Figure 4. During the analysis of IR spectra for certain molecules, the focus was on their main characterization peaks. In Figure 3, kaolinite’s peaks appeared at 3695, 3671, 3655, and 3619 cm^−1^. These peaks appeared due to -OH stretching modes. After DMSO intercalation, new peaks arise at 3661, 3540, 1427, 1402, 1390, and 1318 cm^−1^. Methylation steps draw changes in DMSO-modified kaolinite where 3695 and 3619 cm^−1^ peaks remain visible, but a new peak at 1654 cm^−1^ appears due to OH bending in the system [36]. Both APTMS and CTAC intercalation have an impact on IR spectrums. The peaks at 2918 and 2849 cm^−1^ appear due to -C-H- stretching from aliphatic groups of those intercalating agents [37,38].

In Figure 4 IR spectra for NMF intercalated kaolinites peaks observed at 3695, 3619, 3419, 2905, 1681, 1528, 1419, 1379, and 1237 cm^−1^ where 3419 and 1528 cm^−1^ are characteristic peaks for NMF. At 3419 cm^−1^, the signal indicates that the NMF molecule intercalated with kaolinite through H bonding, and 1528 cm^−1^ refers to the C-H peak in the NMF molecule that arose due to C-H bending [24]. After methylation, significant peaks for NMF at 3419 cm^−1^ disappear, but peaks at 1681 cm^−1^ show traces of NMF, and 1650 cm^−1^ indicated the presence of OH bending from adsorbed water [36]. After intercalation with APTMS and CTAC, it is found that the CTAC intercalated system has sharp peaks at 2918 and 2849 cm^−1^, but for APTMS, a weak signal at 2918 cm^−1^ was observed. This indicates APTMS is less pronounced in the system.

### 3.1. Morphology

**Figure 5:**
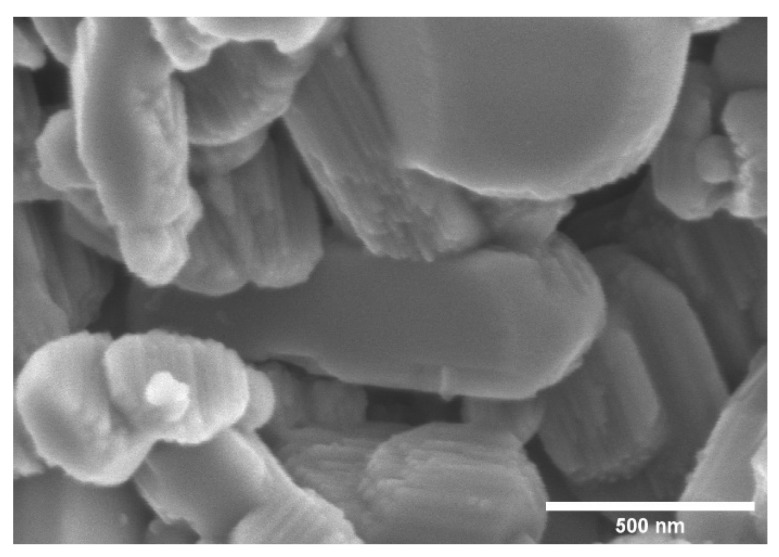
SEM image of 200 meshed kaolinite powder.

**Figure 6:**
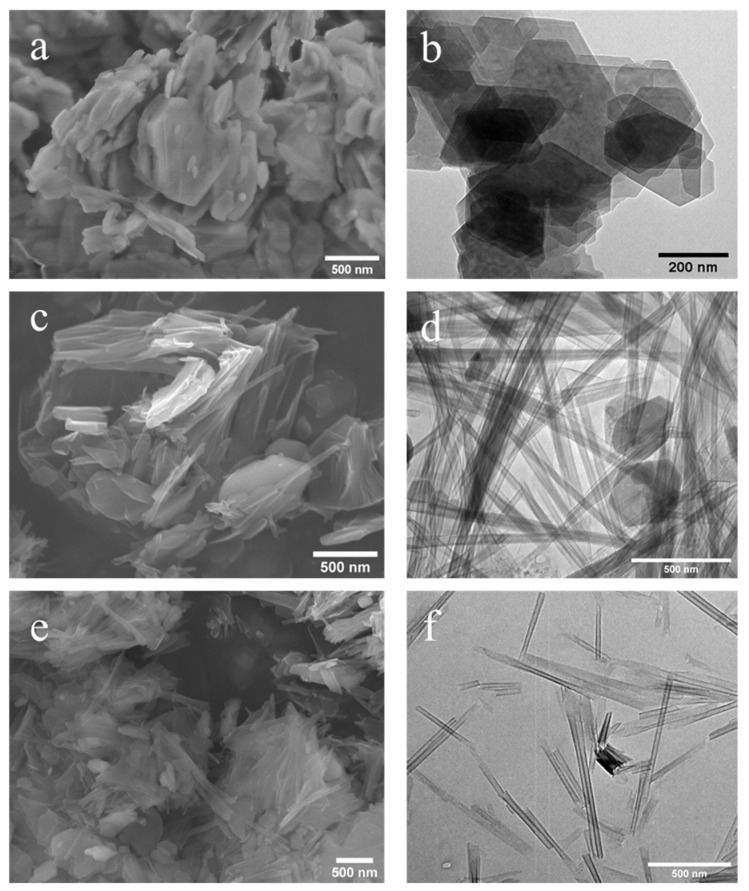
SEM and TEM images of DMSO-modified methylated kaolinite (**a**,**b**); APTMS intercalated NScs-1 (**c**,**d**) and CTAC intercalated NScs-2 (**e**,**f**), respectively. (SEM images (**a**,**c**), and (**e**); TEM images, (**b**,**d**), and (**f**).

**Figure 7:**
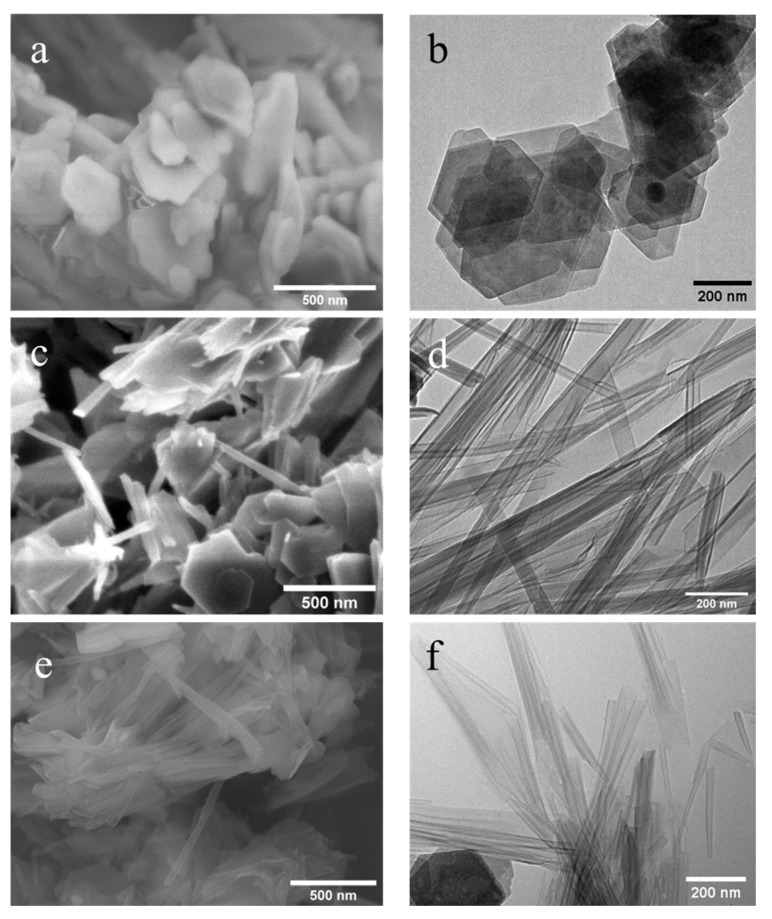
SEM and TEM images of NMF-modified methylated kaolinite (**a**,**b**); APTMS intercalated NScs-3 (**c**,**d**) and CTAC intercalated NScs-4 (**e**,**f**), respectively. (SEM images (**a**,**c**), and (**e**); TEM images, (**b**,**d**), and (**f**).

For the morphological studies of kaolinites, methoxy-modified kaolinites and nanoscrolls were observed by electron microscopy. Figure 5 shows the SEM image of 200 mesh kaolinite powder, which reveals the stacked sheet-like structure of kaolinite. It is also noticeable that smaller (<1 μm) kaolinite particles are present in the starting material. Figure 6 and Figure 7 present both SEM and TEM images of the DMSO and NMF-originated nanoscrolls, respectively. The SEM image of KM-1 (Figure 6a) shows that after methylation, the sample forms thinner plate-like structures compared to that of the bulkier kaolinite precursor (Figure 5) and TEM of KM-1 (Figure 6b) shows after methylation, the crystallites form thin platelets and retain a hexagonal shape. Figure 6c,d show the SEM and TEM images of nanoscrolls (NScs-1), respectively. Nanoscrolls, alongside partially formed scrolls, curled sheets, and small kaolinites nanosheets, are observed.

From the TEM image (Figure 6d), it is found that the scroll walls are multilayered and the scrolls themselves have average inner and outer diameters of 15.2 ± 3.8 and 37.3 ± 8.8 nm, respectively. Figure 6e,f shows, respectively, the SEM and TEM images of nanoscrolls formed by the CTAC intercalation (NScs-2). Here, fully formed nanoscrolls, partially formed nanoscrolls, and small particles (or nanosheets) of kaolinite are observed. TEM images show that the inner and outer diameter of the NSc-2 nanoscrolls are 14.8 ± 4.2 nm and 24.9 ± 6.1 nm, respectively.

Figure 7 represents the SEM and TEM images of NMF-originated nanoscrolls with methylated kaolinites. Figure 7a,b show the SEM of TEM, respectively, of methylated kaolinite, KM-2, and it is found that they also have become a thin-layered structure with a hexagonal shape as seen with the DMSO-originated methylated kaolinite. The SEM images of nanoscrolls formed by APTMS (NScs-3) or CTAC (NScs-4) reveal morphologies consisting of fully formed nanoscrolls, partially formed nanoscrolls, curled nanosheets, and small particles of kaolinite. The images of these nanoscrolls reveals that APTMS intercalated nanoscrolls are multilayered or thick-walled compared to that of CTAC intercalated nanoscrolls, that the average inner diameter of NScs-3 is 13.8 ± 3.3 nm and of NScs-4 is 14.1 ± 3.7 nm, and that the outer diameter of NScs-3 is 35.1 ± 7.7 nm and of NScs-4 is 24.3 ± 4.2 nm.

### 3.2. Surface Area and Porosity Results

**Figure 8:**
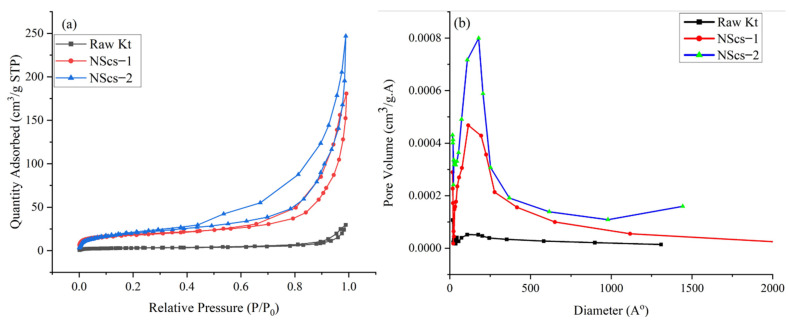
N_2_ adsorption-desorption isotherms (**a**) and pore size distribution histograms (**b**) of untreated kaolinite, NScs-1, and NScs-2.

**Figure 9:**
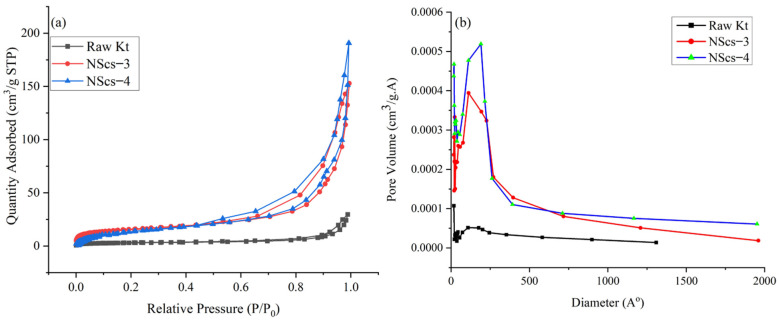
N_2_ adsorption-desorption isotherms (**a**) and pore size distribution histograms (**b**) of untreated kaolinite, NScs-3, and NScs-4.

The surface area of untreated kaolinite and nanoscrolls were characterized by the BET method. The Figure 8 and Figure 9 shows the N_2_ adsorption and desorption isotherms and pore size distribution of different nanoscrolls and raw kaolinites. The isotherms of all nanoscrolls (Figure 8a and Figure 9a) show type IV isotherms, while unreacted kaolinite’s isotherm is type II. The surface areas for the various components varied with processing and, as would be expected, the observed values of the nanoscroll samples are larger than bulk kaolinite powder: kaolinite (10.16 m^2^/g), NScs-1 (63.34 m^2^/g), NScs-2 (73.14 m^2^/g), NScs-3 (54.48 m^2^/g), and NScs-4 (62.28 m^2^/g).

The pore size distributions were determined by the BJH desorption dV/dD pore volume method. Figure 8b and Figure 9b show the pore size distribution for different nanoscrolls compared to the raw kaolinite. From the respective isotherms (Figure 8b and Figure 9b), it is found that NScs-1 has an inner diameter in the range of 11–20 nm, for NScs-2 it is 10–18 nm, for NScs-3 it is 11–23 nm, and for NScs-4 it is 11–20 nm. In Table 1, the average inner diameter obtained from TEM observation and BJH desorption is presented.

## 4. Discussion

The three-step rapid synthesis of kaolinite NScs highlighted in this report utilizes microwave processing. It is found that not only the initial DMSO/NMF and final APTMS/CTAC intercalation steps readily occur but that the more difficult methylation step is greatly accelerated (reduced to 3.5 h). This 14-h overall process is the fastest reported to date and leads to high-quality Kt NScs. Table 2 compares the processing times with other reported methods for the synthesis of kaolinite nanoscrolls. The processes mainly contain three general steps where methylation dominates the total duration of the synthesis. The microwave method applied in this experiment reduces the methylation duration and decreases overall synthesis time. The surface area obtained from the BET measurement indicates that this experiment’s outcome is in good agreement with previous literature for CTAC based NScs [17]. It is noteworthy that while in most respects nanoscrolls originating from DMSO and NMF are similar, the ones from DMSO-CTAC modification were in general better dispersed.

The d spacing (0.69 nm) of kaolinite powder used as a precursor in this experiment is in good agreement with previous reports (~0.72 nm) [16]. After intercalation with DMSO, it was found that the interlayer spacing increased up to 1.06 nm [26,40]. When NMF was used as the primary intercalating agent, the increment was 0.33 nm, which was also observed by Kelleher et al. and Komori et al. [24,41]. It is important to note that the XRD samples are prepared by drying the samples at 70 °C for 24 h, which indicates that the primary intercalant is thermally stable under mild heating. After methylation, it was found that the dried methylated kaolinite sample interlayer spacing decreased to 0.82 nm, which is a general phenomenon [42].

After treatment with APTMS on methylated kaolinite, the sample becomes amorphous with ultrasonication irrespective of their primary intercalant. This is highlighted in the broadness of the peaks (Figure 1d,e and Figure 2d,e). When CTAC treatment and ultrasonication were carried out on methylated-modified kaolinite, a trace of CTAC remained in the DMSO-originated kaolinite. According to the findings of Komarneni et al., ultrasonication is responsible for the amorphous nature of the product [18]. After the final intercalation, the presence of d-spacing of 0.69 nm as seen by XRD indicates that the trace of unreacted kaolinite remained in the samples.

IR analysis provides further insight into the formation of kaolinite NScs. Unreacted kaolinite’s signature peaks, which appear in the 3600–3700 cm^−1^ region, were weakened in intensity (Figure 3) after intercalation with DMSO. Besides this, the appearance of 3661, 3540, and 3505 cm^−1^ peaks indicate the formation of hydrogen bonds between kaolinite and DMSO [20,27,43]. After methylation of DMSO, 3540 and 3505 cm^−1^ signals disappear, consistent with methylation.

When NMF is intercalated in bulk kaolinite the intensity of the 3695 cm^−1^ band becomes weak as expected. Additionally, 3419 cm^−1^ and 2905 cm^−1^ bands appear due to NMF N-H and C-H stretching, respectively. When methylation occurs to NMF, these two bands disappear as expected, due to the displacement of NMF in the structure [24,36].

On treatment of methylated kaolinite with APTMS, washing, and ultrasonication in toluene, irrespective of their primary intercalant, the dried samples show signals at 2918 and 2849 cm^−1^, which have appeared due to C-H stretching of -CH_2_ and -CH_3_ aliphatic groups, respectively, from APTMS [38]. Upon intercalation with CTAC with methylated kaolinite, similar peaks appeared at 2918 and 2849 cm^−1^ due to C-H stretching from CTAC [37].

The transformation of stacked kaolinite to nanoscrolls can be appreciated through the monitoring structural variations after each processing step. From Figure 6, it is visible that kaolinite starts as well-formed plate-like crystals until the DMSO or NMF intercalated is methylated (Figure 6a and Figure 7a), which leads to the exfoliation of nanosheets. Subsequent treatment with APTMS or CTAC leads to well-formed nanoscrolls. Interestingly, it is found that, on average, APTMS intercalated nanoscrolls have more outer layers (turns) than CTAC intercalated nanoscrolls and that their inner and outer diameters are different from each other. Kuroda et al. suggested that the size variation is related to the identity of the final intercalating agent, such that APTMS intercalation favors larger sheets of kaolinite and forms nanoscrolls with a larger outer diameter compared to CTAC [17].

The surface area and porosity analysis reveal the materials size, porosity, and shape. The surface area of raw kaolinite and different nanoscrolls also has coherency with previous reports. Especially, it is found that when CTAC is used as the final intercalating agent compared to the APTMS intercalated nanoscrolls, the surface area is larger [17,27,39]. Additionally, the isotherm obtained for kaolinite infers that the raw materials were mainly macroporous with the trace of few mesoporous and microporous powders [44]. On the other hand, the isotherm obtained for nanoscrolls is type IV with an H2 loop. This indicates that the final samples obtained from this experiment were mainly meso to microporous materials [45]. The finding of the inner diameters of NScs from BJH method are in good agreement with the TEM measurements of inner diameters of NScs.

## 5. Conclusions

The microwave method presented here significantly reduces processing times in the synthesis of kaolinite NScs. Intercalation with both DMSO and NMF were successful under microwave radiation, and the methylation step was especially efficient. After intercalation and ultrasonication with APTMS or CTAC, the processing produced high-quality nanoscrolls. Overall, this indicates that microwave processing is an effective advance in the rapid production of kaolinite nanoscrolls.

## Figures and Tables

**Table 1:** Inner diameter and surface area of different nanoscrolls.

Nanoscrolls	Average Inner Diameter (TEM Observations) (nm)	Average Inner Diameter (BJH Method) (nm)
NScs-1	15.2 ± 3.8	16.4
NScs-2	14.8 ± 4.2	13.5
NScs-3	13.8 ± 3.3	15.3
NScs-4	14.1 ± 3.7	15.1

**Table 2:** Comparison of processing times for the synthesis of kaolinite NScs.

Most Time-Consuming Step	Method	Duration (h)	No. of Steps	Total Duration (h)	Surface Area of NScs (m^2^/g)	Reference
Methylation	Stirring	120	3	>168	71	Kuroda et al. [17]
Methylation	Stirring	>168	3	240	32.6	Yuan et al. [15]
Methylation	Solvothermal	24	3	84	……	Xu et al. [39]
Methylation	Washing	24	5	96	100.7	Li et al. [18]
Intercalation with DMSO or Urea	Aged in a closed vessel	168 or 456	3 or 2	216 or 480	……	Mako et al. [19]
Intercalation With DMSO	Stirring	24	3	72	39.6	Qu et al. [27]
Intercalation with DMSO	Microwave	5	3	14	73.1	This work

## Data Availability

The data presented in this study are available on request from the corresponding author.
